# Ketogenic Diet Suppressed T-Regulatory Cells and Promoted Cardiac Fibrosis via Reducing Mitochondria-Associated Membranes and Inhibiting Mitochondrial Function

**DOI:** 10.1155/2021/5512322

**Published:** 2021-04-19

**Authors:** Jun Tao, Hao Chen, Ya-Jing Wang, Jun-Xiong Qiu, Qing-Qi Meng, Rong-Jun Zou, Ling Li, Jun-Gang Huang, Zong-Kai Zhao, Yu-Li Huang, Hai-Feng Zhang, Jun-Meng Zheng

**Affiliations:** ^1^Department of Cardiovascular surgery, Sun Yat-Sen Memorial Hospital, Sun Yat-sen University, Guangzhou, China; ^2^Department of Gastroenterology, Guangdong Provincial People's Hospital, Guangdong Academy of Medical Sciences, Guangzhou, China; ^3^Department of Otorhinolaryngology-Head and Neck Surgery, Sun Yat-sen Memorial Hospital, Sun Yat-sen University, Guangzhou, China; ^4^Department of Orthopedics of Guangzhou Red Cross Hospital, Medical College, Jinan University, Guangzhou, China; ^5^Heart Center, Guangdong Provincial Key Laboratory of Research in Structural Birth Defect Disease, Guangzhou Women and Children's Medical Center, Guangzhou Medical University, Guangzhou, China; ^6^Department of Cardiology, Shunde Hospital, Southern Medical University, Foshan, China; ^7^Department of Cardiology, Sun Yat-sen Memorial Hospital, Sun Yat-sen University, Guangzhou, Guangdong, China; ^8^Department of surgery, Kiang Wu Hospital, Macau SAR, China

## Abstract

Ketogenic diet (KD) is popular in diabetic patients but its cardiac safety and efficiency on the heart are unknown. The aim of the present study is to determine the effects and the underlined mechanisms of KD on cardiac function in diabetic cardiomyopathy (DCM). We used db/db mice to model DCM, and different diets (regular or KD) were used. Cardiac function and interstitial fibrosis were determined. T-regulatory cell (Treg) number and functions were evaluated. The effects of ketone body (KB) on fatty acid (FA) and glucose metabolism, mitochondria-associated endoplasmic reticulum membranes (MAMs), and mitochondrial respiration were assessed. The mechanisms *via* which KB regulated MAMs and Tregs were addressed. KD improved metabolic indices in db/db mice. However, KD impaired cardiac diastolic function and exacerbated ventricular fibrosis. Proportions of circulatory CD4^+^CD25^+^Foxp3^+^ cells in whole blood cells and serum levels of IL-4 and IL-10 were reduced in mice fed with KD. KB suppressed the differentiation to Tregs from naive CD4^+^ T cells. Cultured medium from KB-treated Tregs synergically activated cardiac fibroblasts. Meanwhile, KB inhibited Treg proliferation and productions of IL-4 and IL-10. Treg MAMs, mitochondrial respiration and respiratory complexes, and FA synthesis and oxidation were all suppressed by KB while glycolytic levels were increased. L-carnitine reversed Treg proliferation and function inhibited by KB. Proportions of ST2L^+^ cells in Tregs were reduced by KB, as well as the production of ST2L ligand, IL-33. Reinforcement expressions of ST2L in Tregs counteracted the reductions in MAMs, mitochondrial respiration, and Treg proliferations and productions of Treg cytokines IL-4 and IL-10. Therefore, despite the improvement of metabolic indices, KD impaired Treg expansion and function and promoted cardiac fibroblast activation and interstitial fibrosis. This could be mainly mediated by the suppression of MAMs and fatty acid metabolism inhibition *via* blunting IL-33/ST2L signaling.

## 1. Introduction

Diabetic individuals suffer from much more cardiovascular complications than nondiabetic patients. Besides coronary artery disease, ventricular damage is also frequently evident in diabetic patients, which refers to a term of “diabetic cardiomyopathy” (DCM). The incidence of DCM greatly contributes to heart failure, which is the main complication leading to life and health loss in diabetic patients. In clinical aspect, DCM is characterized by the impairment of diastolic function while the systolic function remained unchanged, especially in the early stage of the disease. From the microcosmic view, cardiomyocyte hypertrophy and interstitial cardiac fibrosis are the fundamental changes [1].

Ketogenic diet (KD) is a very low-carbohydrate, high-fat diet that typically includes plenty of meats, eggs, cheeses, fish, and nuts, which is a therapy to epilepsy in clinical practice. It has been shown to improve blood sugar control for patients with type 2 diabetes (T2DM) and thus widely sued in these patients. Moreover, it has been proposed to be a preventive or therapeutic strategy for metabolic disorders [2]. However, on the other hand, it has been linked to vascular insult recently [3]. In the field of cardiac remodeling, KD is reported to be associated with improved cardiac function, cardiomyocyte survival, and attenuated cardiac fibrosis in both T2DM and aging mice [4, 5]. In contrast, ketogenic diet was shown to be associated with cardiac remodeling in hypertensive rats [6]. Besides, *in vitro* study directly showed that the ketone body strengthened cardiac fibroblast activation induced by transforming growth factor-*β*1, which is a hallmark of the enhanced interstitial fibrosis [6]. Therefore, more studies are needed in order to clarify the detailed roles of KD on DCM.

Besides the heart itself, KD could modify the innate immune system that is closely related to the development of DCM. For example, KD regulates the T cell subset and group 2 innate lymphoid cells [7–11]. However, the alterations of KD on the innate immune system during DCM are also unknown. On the other hand, the ketone body was found to be associated with enhanced mitochondrial respiratory function and substrate metabolism, [12, 13] which is crucial to T cell subset differentiation and function [14, 15]. Among various factors critically involved in mitochondrial respiratory function and substrate metabolism, mitochondria-associated endoplasmic reticulum membranes (MAMs) are an important one [16, 17].

IL-33 acts through its receptor ST2. The membrane ST2 (ST2L) mediates the biological effects of IL-33 while the soluble ST2 (sST2) acts as the decoy receptor that restricts the effects of IL-33 [18]. We recently demonstrated that interleukin-33 (IL-33) protected diabetic mice from DCM and reduced cardiac fibrosis [19]. However, the role of changes of endogenous IL-33/ST2L signaling and their potential roles in Tregs and KD-treated diabetes are unknown.

Thus, to link KD and DCM development, we demonstrated the effects of KD on cardiac fibrosis of DCM and Tregs. We found that KD impaired cardiac function and addressed the roles of MAMs, Treg substrate metabolism, and IL-33/ST2L signaling in this process.

## 2. Methods

### 2.1. Animals and Cells

Male C57BLKsJ-db/db mice were used to establish the spontaneous T2DM and DCM as our previous report [19]. Age-matched male C57BLKsJ-m/m mice were used as the control [19]. Mice were kept in a 12 h light/dark cycle with free access to chow and water. Eight-week-old db/db mice were randomly assigned to receive either regular chow diet (RD, % of total kcal, 18% protein, 65% carbohydrate, and 17% fat) or KD (% of total kcal, 10% protein, <1% carbohydrate, and 89% fat) for 12 weeks. The nutritional composition of the ketogenic diet was shown in Table 1. After 12 weeks of treatment, mice were subjected to echocardiography and invasive hemodynamic assessment. After that, mice were anesthetized by intraperitoneal injection of ketamine (80 mg/kg body weight) and xylazine (5 mg/kg body weight) and then were sacrificed. The left ventricular was acquired and cut, which were then fixed in 4% paraformaldehyde or cryopreserved in liquid nitrogen according to different further assessments. All the animals were purchased from the Model Animal Research Center of Nanjing University. All the animal procedures were conformed to the Guide for the Care and Use of Laboratory Animals published by the US National Institutes of Health and were approved by the Institutional Animal Care and Use Committee of Sun Yat-sen University.

Peripheral blood mononuclear cell (PBMC) or CD4^+^ T cells were used in the *in vitro* study. PBMCs were isolated by the density gradient centrifugation method with Ficoll (TBD science, density: 1.079 g/ml, Tianjin, China) as previously reported [20]. Briefly, for isolation, whole blood samples were collected in the heparin anticoagulation condition. Blood samples were diluted with the same volume of PBS, which were then overlaid on Ficoll. PBMCs were isolated in the middle layer after centrifugation, which were collected for further analysis. For CD4^+^ T cells and CD4^+^CD25^+^Foxp3^+^ Treg isolations, the mouse spleen were obtained and splenocytes were retrieved by mechanical dissociation of the spleen on 70 *μ*m Cell strainer filter (BD Falcon™, New Jersey, USA) [21]. Cell suspensions were then transferred for isolation using the commercially available kits (EasySep™, StemCell Tech, Vancouver, Canada) under the manufacturer's instructions. Isolated CD4^+^ T cells were then resuspended and cultured in the RPMI-1640 medium (Thermo Fisher, Massachusetts, USA) containing 10% FBS (Thermo Fisher, Massachusettsm, USA), 2 mM L-glutamine (Sigma-Aldrich, Missouri, USA), 1% penicillin/streptomycin, and 50 *μ*M mercaptoethanol (Sigma-Aldrich, Missouri, USA). The ketone bodies used in the current study are 25 mM *β*-hydroxybutyrate (Sigma-Aldrich, Missouri, USA).

### 2.2. Echocardiogram and Invasive Hemodynamic Assessments

Echocardiogram and invasive hemodynamic assessment were used to assess *in vivo* cardiac function. Animals were anesthetized as mentioned above. Left ventricular ejection function (LVEF) and left ventricular end diastolic dimension (LVEDd) were measured by the Visual Sonics Echo System (Vevo2100, VisualSonics, Toronto, Canada) and MicroScan Transducer (MS-400, 30 MHz, VisualSonics, Toronto, Canada) [19].

Hemodynamic assessment was performed before sacrifice following anesthesia according to the previous report [22]. Briefly, a percutaneous puncture of the carotid was performed under sterile conditions. After the Millar catheter (Mikro-Tip®, Millar Instruments, Houston) insertion, heparin (100 IU/kg, iv) was administered. ±*dp*/*dt*_max_ was obtained by MPVS Ultra™ pressure-volume unit and PowerLab data acquisition system (ADInstruments, Sydney, Australia). All measurements were performed after the confirmation of hemodynamic stability for 2 min. An average of 3 respiratory cycles was used for analysis.

### 2.3. Isolation of MAMs, Western Blot, and Polymerase Chain Reaction (PCR)

MAMs were isolated using the modified protocol according to the previous reports [23]. Briefly, Tregs were ultrasonically fragmented, which were centrifuged for 5 min at 750 g for 2 times. Supernatants were collected and were subjected to high-speed centrifugations (10 min for 9000 g ×1 and 10000 g ×2). Pellets were collected and resuspended in solution (250 mM mannitol, 0.5 mM EGTA, and 5 mM HEPES; pH 7.4), which were then subjected to ultraspeed centrifugation (100000 g, 30 min). The middle layers were collected for centrifugations (6300 g, 10 min), and the supernatants were collected for final centrifugations for 1 h at 100000 g. All centrifugations were done in 4°C. Protein quantification analysis using BCA methods according to the manufacturer's instructions (Cell Signaling Technology, Massachusetts, USA) was adopted to represent the amounts of MAMs. The pure mitochondria isolated using the commercially available kit (Thermo Fisher, Massachusettsm, USA) as our previous report [24] were used as the inner control.

Western blot was performed as our previous report [25]. Briefly, samples were collected and washed. Totally proteins were extracted and quantified using the BCA methods. The amount of 60 *μ*g total proteins was subjected to SDS-PAGE electrophoresis, which was then transferred to a PVDF membrane (Millipore, Massachusetts, USA). Finally, PVDF membranes were incubated with respective antibodies (all were from Abcam, Cambridge, UK) and electrochemiluminescence (Millipore, Massachusetts, USA) was added to visualize bands. The ImageJ software was used for densitometry measurements.

To assess mtDNA copy number, quantified PCR was performed. Briefly, DNA was extracted and the short segment of mtDNA was amplified, which was compared to the amplification of the short segment of nuclear DNA (*β*-globin) [26]. Primers used were also listed in the previous report [26].

### 2.4. Masson's Trichrome Staining and Hydroxyproline Assay

Masson's Trichrome staining and hydroxyproline assessment were used to evaluate contents of ventricular collagen. For Masson's Trichrome staining, ventricular tissues fixed in 4% paraformaldehyde were embedded in paraffin, which were then subjected to cut into 5 *μ*m slices. Slices were deparaffinized and rehydrated. After that, slices were stained using Trichrome Stain (Masson) kit (Sigma-Aldrich, Missouri, USA) under the manufacturer's instructions. Collagen, muscle, and nuclei are stained in blue, red, and black, respectively.

### 2.5. Flow Cytometry

Flow cytometry was used to detect fatty acid absorption, intracellular fatty acid, and CD4^+^CD25^+^Foxp3^+^ Tregs. For fatty acid absorption, BODIPY (BODIPY 503/512, Thermo Fisher Scientific, Massachusettsm, USA) was used. It is a green fluorescent fatty acid, which could be used to monitor fatty acid absorption by cells [27]. For the detection of intracellular fatty acid, another BODIPY (BODIPY 505/515, Thermo Fisher Scientific, Massachusettsm, USA), a cell membrane permeant fluorophore and a stain for natural lipids, was used. Flow cytometry was used to detect the fluorescence by BODIPY. All the flow cytometry data were acquired by LSR Fortessa flow cytometer (BD, New Jersey, USA) and were analyzed with FlowJo software (Version 10, Tree Star Inc.).

### 2.6. Cell Viability and Treg Cytokine Assessment

Cell viability of Tregs were reflected by CCK-8 levels determined using Cell Counting Kit-8 (CCK-8, Dojindo Molecular Technologies, Kumamoto, Japan). Interleukin-4 (IL-4) and interleukin-10 (IL-10) are the main cytokines represented by Treg functions. They were determined by enzyme-linked immunosorbent assay (ELISA). Procedures of CCK-8 measurements and levels of IL-4 and IL-10 determination were performed under the manufacturer's instructions (kits of ELISA, R&D Systems, Minnesota, USA).

### 2.7. Seahorse Analysis

Oxygen consumption rate (OCR) was measured using Seahorse XF96 (Agilent, Delaware, USA) as our previous report [28]. Briefly, cells were cultured in the working medium (Seahorse XF basal medium, Catalog, 103335-100, Agilent, Delaware, USA). Basal oxidative phosphorylation (OXPHOS) and ATP-linked OXPHOS were calculated from the OCR profile to represent mitochondrial respiratory function.

### 2.8. Statistical Analysis

Normally distributed data are expressed as mean ± SEM. One-way ANOVA, followed by SNK test for multiple post hoc comparisons, was adopted to test the statically differences among groups. A *P* value < 0.05 indicated the statistical significance. All the statistical analyses were done with the OriginLab software (version2019b, Massachusettsm, USA).

## 3. Results

### 3.1. KD Promoted Cardiac Fibrosis and Worsened Diastolic Function despite Metabolic Improvements

We examined the effects of KD on metabolic dysfunction in mice first. Metabolic indices were listed in Table 2. In summary, body weight, heart weight, serum fasting glucose, and cholesterols were improved by KD. Besides, serum insulin was decreased, and the insulin sensitivity, as indicated by HOMA-IR, was improved by KD (Table 2).

**Figure 1:**
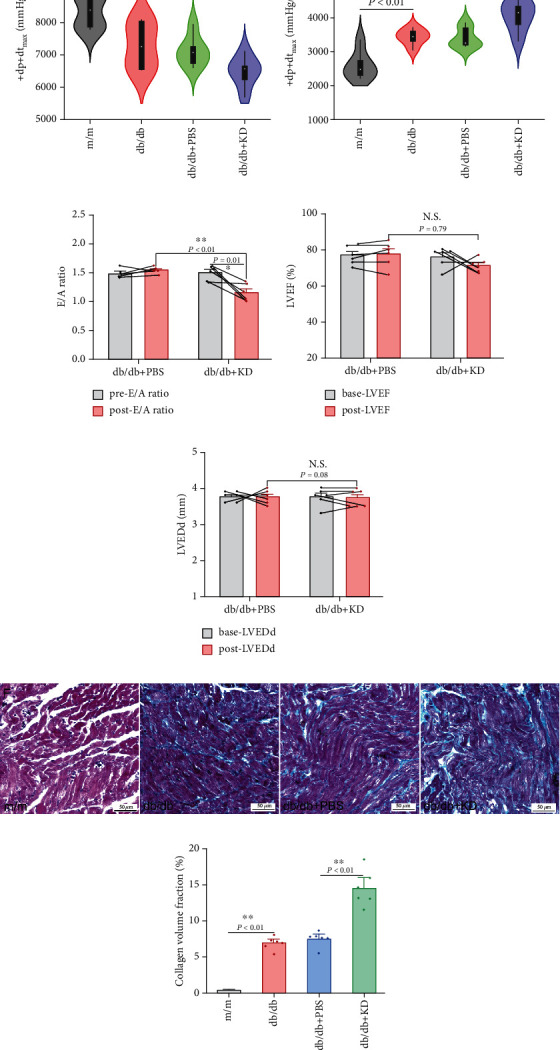
Effects of ketogenic diet on cardiac functions, left ventricular size, and collagen contents. Cardiac functions were represented as +*dp*/*dt*_max_ (a), −*dp*/*dt*_max_ (b), E/A ratio (c), LVEF (d) and LVEDd (e). Left ventricular size was represented as LVEDd. Collagen contents were represented as Masson's Trichrome staining (f) quantified as collagen volume fraction (g). KD: ketogenic diet; LVEF: left ventricular ejection fraction; LVEDd: left ventricular end-diastolic diameter.

We next assessed ventricular performance and function in mice. Unexpectedly, as results measured by the hemodynamic methods, cardiac contractile function (*dp*/*dt*_max_, Figure 1(a)) was mildly decreased while the diastolic function (−*dp*/*dt*_max_, Figure 1(b)) were obviously reduced in KD-treated mice. Results from UCG measurements showed that mice treated with KD, compared with those either with PBS control or with pre-KD treatment, showed a decreased E/A ratio (Figure 1(c)), while nonsignificant changes of LVEF (Figure 1(d)) and LVEDd (Figure 1(e)) could be observed.

Diastolic dysfunction may be due to cardiomyocyte hypertrophy and/or declined ventricular compliance. However, heart weight was not significantly different among mice with different treatments, which might not support the significant role of hypertrophy during this process. Interstitial fibrosis is the key for ventricular compliance; therefore, we sought for the evidence of cardiac interstitial fibrosis in mice. As expected, Masson's Trichrome staining revealed that ventricular collagen levels were much more obvious in mice fed with KD (Figures 1(f) and 1(g)). All these results showed that KD worsened diastolic function and promoted interstitial fibrosis despite improvements of serum glucose.

### 3.2. KD Resulted in Circulating Treg Reduction and Cardiac Fibroblast Activation

**Figure 2:**
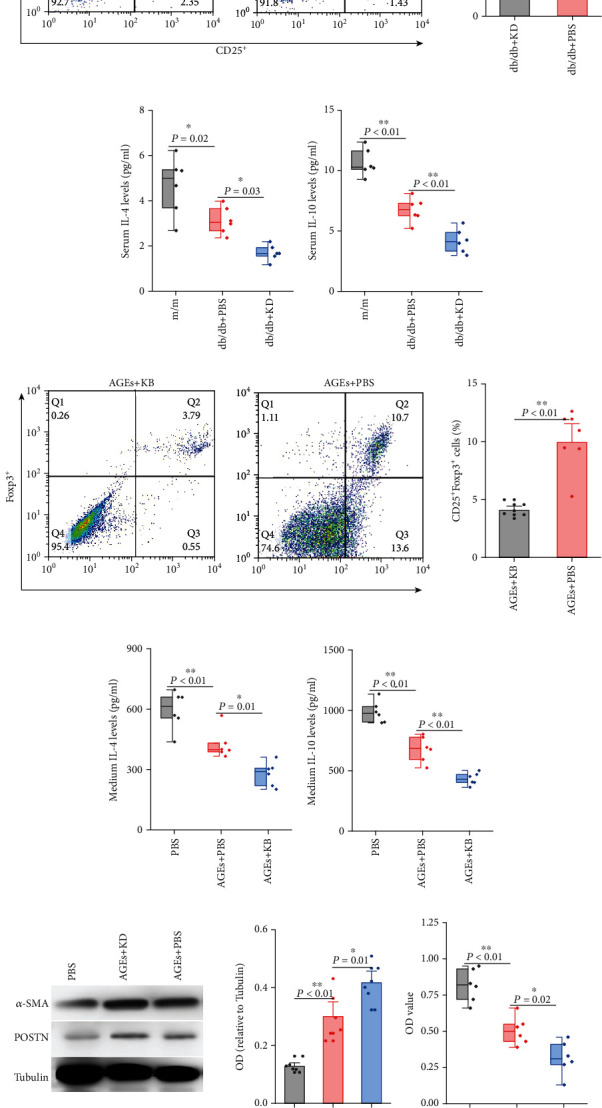
Ketogenic diet suppressed Treg functions which promoted cardiac fibroblast activation. Effects of ketogenic diet on Treg proportions in whole blood cells (a) and serum Treg cytokines (b, c). Effects of ketogenic diet on Treg proportions in CD4^+^ T cells derived from the spleen (d) and medium Treg cytokines (e, f). Effects of spleen-derived CD4^+^CD25^+^Foxp3^+^ Treg cultured medium from different treatments on activation markers (g) and cell vitality (h). *P* values were derived either from the *t*-test (a, d) or the ANOVA followed by SNK for multiple comparisons (b, c, e–h).

The reason of the exacerbated cardiac fibrosis elicited by KD is unknown, and Tregs played an important role in cardiac fibrosis. We therefore continued to explore the changes of Tregs upon KD treatment. As shown in Figure 2(a), KD resulted in a reduced proportion of CD4^+^CD25^+^Foxp3^+^ cells in whole blood cells. Besides, serum levels of Treg cytokines, IL-4 and IL-10, were also decreased in KD-treated mice (Figures 2(b) and 2(c)). *In vitro*, KB, compared with PBS control, significantly reduced the proportion of CD4^+^CD25^+^Foxp3^+^ cells induced from spleen CD4^+^ T cells (Figure 2(d)). IL-4 and IL-10 productions in supernatants of these cells were also decreased (Figures 2(e) and 2(f)).

The results shown in Figure 1 supported the role of interstitial fibrosis in DCM diastolic dysfunction, and we therefore focused on the activation of cardiac fibroblasts. However, the roles of suppressing Treg differentiation from T cells by KD/KB on cardiac fibroblasts activation is unknown and we therefore continued to explore them. Results found that cultured medium from KB-treated Tregs dramatically activated cardiac fibroblasts, as manifested by *α*-SMA and POSTN expressions assessed by Western blotting (Figure 2(g)). Proliferation assays also supported cardiac fibroblast activation. Compared with the cultured medium from Tregs that received PBS treatment, CCK-8 levels were increased in cells treated with the medium from KB-treated Tregs (Figure 2(h)). All these results supported that the regulation on Treg expansion and function are critical in cardiac fibroblast activation.

### 3.3. KB Orchestrated FA and Glycolysis Metabolisms and Suppressed Tregs

The mechanism that KB regulates Tregs is unknown, and the metabolisms of fatty acid and glucose are crucial for Treg expansion and function. Therefore, we continued to investigate the changes of fatty acid and glucose metabolism under KB treatment.

**Figure 3:**
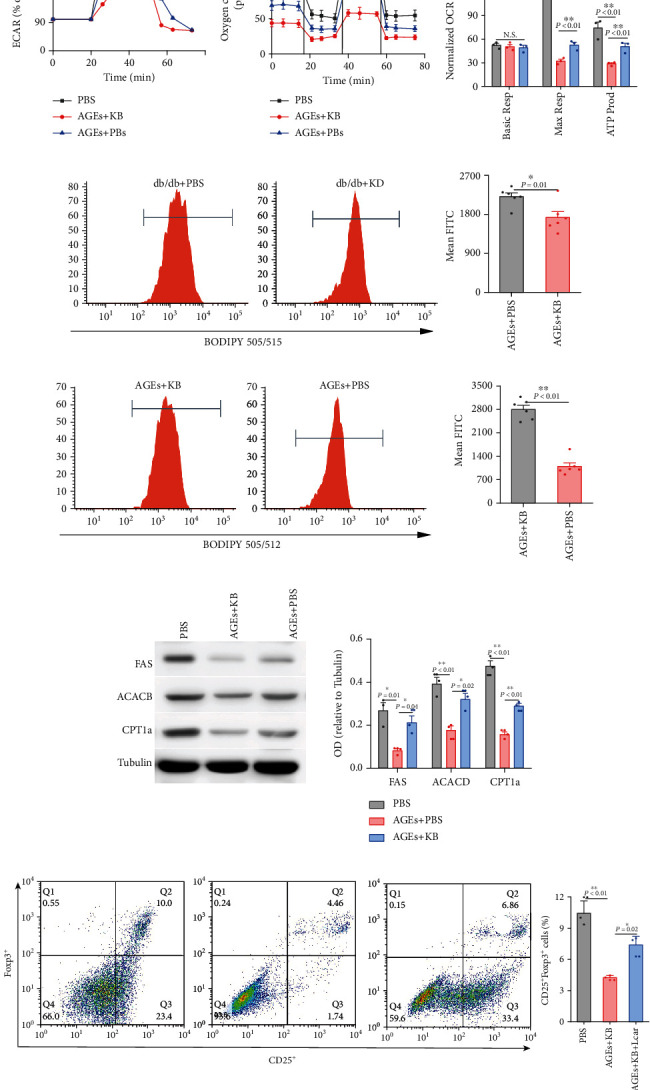
Effects of KB on glucose and FA metabolism in Tregs. Effects of KB on extracellular acidification rates (a) and oxygen consumption (b, c) in CD4^+^CD25^+^Foxp3^+^ cells derived from the spleen. Effects of KB on intracellular FA contents (d) and absorption (e) in CD4^+^CD25^+^Foxp3^+^ cells. Effects of KB on key genes involved in FA synthesis and oxidation (f). Effects of KB on CD25^+^Foxp3^+^ cells derived from CD4+ T cells (g). *P* values were derived either from the ANOVA followed by SNK for multiple comparisons (a–c, f, g) or the *t*-test (d, e). Basic Resp: basic respiration; Max Resp: maximum respiration; ATP Prod: ATP production.

We investigated the levels of glycolysis and mitochondrial respiration first. As shown in Figure 3(a), ECAR was increased in cells treated with KB, indicating the increased levels of glycolysis. However, OCR was decreased in cells administered with KB, showing a decreased level of OXPHOS (Figures 3(b) and 3(c)). Accordingly, ATP productions were also reduced (Figure 3(c)).

As for intracellular fatty acid contents, Tregs were separated from the spleen and intracellular fatty acid was determined by BODIPY, a membrane-permeable fluorophore for lipids. As shown in Figure 3(c), compared with regular diet mice, Tregs from KD-treated mice represented a less BODIPY staining (Figure 3(d)), indicating a reduced level of intracellular fatty acid. We next tested whether the absorption of fatty acid by Tregs was also decreased. Unexpectedly, we found that KB treatment resulted in a mildly increased green fluorescence, indicating an upregulated fatty acid absorption (Figure 3(e)). We therefore continued to explore whether, instead, intracellular FA metabolic routes were involved in lipid accumulation and Treg proliferation and function. FA synthesis and oxidation were analyzed, respectively.

The expressions of FA synthesis rate-limiting enzymes, FAS and ACACB, were detected by Western blotting. As shown in Figure 3(f), both the expressions of FAS and ACACB were moderately decreased upon KB treatment. CPT1a is the critical enzyme of mitochondrial FA oxidation; the expressions of which were also investigated. Unexpectedly, CTP1a expressions were decreased to a more extent compared with those of ACACB (Figure 3(f)). Therefore, we continued to explore the role of decreased FA oxidation in Treg suppression by using L-carnitine to facilitate FA oxidation. As shown in Figure 3(g), L-carnitine significantly increased Treg proportions. All these results indicated that FA oxidation inhibition is the key event in KB-induced Treg suppression.

### 3.4. KB Dismissed MAMs and Respiratory Complexes in Tregs *In Vitro*

**Figure 4:**
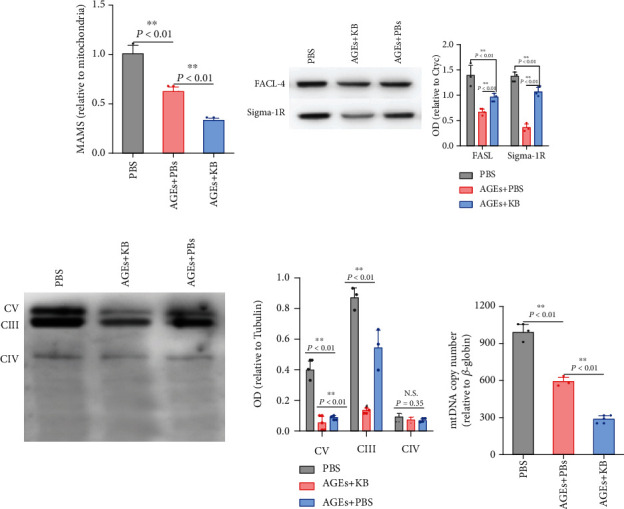
Effects of KB on MAMs (a, b) and mitochondrial respiratory complex (c) contents and mitochondrial number (d). MAMs: mitochondria-associated endoplasmic reticulum membranes; FASL-4: long-chain fatty-acid CoA synthases-4; Sigma-1R: Sigma-1 receptor; OD: optical density; Ctyc: cytochrome C; CV: complex V; CIII: complexes III; CIV: complexes. Other abbreviations were the same as the above.

The mitochondria are the main organelle that FA oxidation occurs. Thus, the above results implied the role of mitochondrial respiration in KB-dependent Treg regulation. MAMs are the key structure that controls mitochondrial respiratory function, and we therefore examined the alterations of MAMs first. As shown in Figure 4(a), the reduction of MAMs was found in AGEs-treated Tregs, as determined by the quantitative analysis of MAM protein levels relative to those of pure mitochondria. Moreover, compared with AGEs alone, MAMs were further decreased in the presence of KB. The MAM proteins, Sigma-1 receptor, and FACL4, as determined by Western blot were also decreased upon KB treatment (Figure 4(b)).

Mitochondrial respiratory complexes are the main proteins that charge OXPHOS, and we also investigated the changes of these respiratory complexes. As shown in Figure 4(c), complexes I, II, and IV were barely detected in all Tregs. Complex III were mostly expressed, followed by complex IV. Both the contents of complexes III and V were decreased by AGEs, which were further decreased in the presence of KB (Figure 4(c)).

Besides MAMs and respiratory complexes, mitochondrial quantity was also examined. As shown in Figure 4(d), Tregs under KB treatment represented less mitochondrial DNA, which supported the reduction of intracellular mitochondrial contents. All these results showed that both mitochondrial functions were significantly impaired in AGEs-treated Tregs, which were exacerbated in the presence of KB.

### 3.5. Attenuation of IL-33/ST2L Signaling Is Essential in KB-Induced MAM Reduction and Treg Inhibition

Despite the fact that the suppression of MAMs could explain the effects of KB on FA oxidation and mitochondrial respiration, it is still unclear how KB inhibits MAMs. The transmembrane receptor, ST2L, is crucial in Treg expansion and function, and we therefore detected the alterations of ST2L in Tregs under KB treatment.

**Figure 5:**
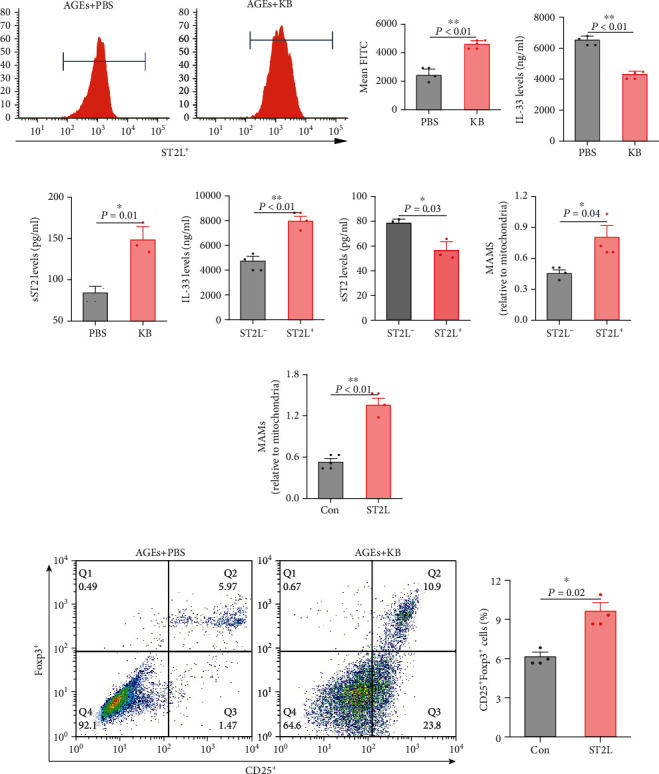
Effects of KB on IL-33/ST2L signaling and its role in KB-induced MAMs and Treg alterations. Flow cytometry detected the changes of membrane ST2L expression in CD4^+^CD25^+^Foxp3^+^ Tregs (a). Cultured medium levels of IL-33 and sST2 PBS-/KB-treated Tregs (b, c). Cultured medium levels of IL-33 and sST2 and MAMs in membrane-ST2L-negative/positive Tregs (d, e). Reinforcement of membrane-ST2L expression on Treg MAMs and Treg proportions in CD4^+^ T cells (f, g, h). sST2: soluble ST2; ST2L^−^: ST2L^−^ Tregs; ST2L^+^: ST2L^+^ Tregs; Con: control vector; ST2L: ST2L vector. Levels of IL-33 were detected by ELISA after NP-40 treatment.

As shown in the results of flow cytometry in Figure 5(a), the proportions of ST2L^+^ Tregs were obviously decreased in KB-treated Tregs. We tried to examine the supernatant contents of IL-33, the only ST2L ligand discovered, which could also activate Tregs, but it was hardly to be detected in the supernatants of Tregs unless NP-40 was added (Figure 5(b)). However, levels of the decoy receptor of IL-33 and sST2, despite the expression levels of which being relatively low, were statistically significantly increased in the supernatant of Tregs treated by KB (Figure 5(c)). Besides, it also should be noted that IL-33 and sST2 productions were even less in ST2L^−^ Tregs (Figures 5(d) and 5(e)). These results showed that KB suppressed IL-33/ST2L signaling in Tregs.

We continued to ask whether the depressed IL-33/ST2L signaling mediated KB-induced MAM suppression and Treg expansion. Expressions of ST2L in Tregs were restored, and the quantities of Tregs and their MAMs were examined. Results found that, compared with the vector control, reinforcement of ST2L in Tregs prevented the loss of MAMs (Figure 5(f)) in KB-treated CD4^+^CD25^+^Fopx3^+^ Tregs (Figure 5(g)). All these results indicated that the depressed IL-33/ST2L signaling is crucial for KB-induced MAM reduction and Treg suppression.

## 4. Discussion

The potential benefits of KB were recognized recently, and the adoption of this diet style is becoming more and more popular. However, the safety and efficiency of KB in the heart function during diabetes are still under sharp debating. In the present study, we demonstrated that KB, despite improved metabolic profiles, resulted in worse heart function and more severe ventricular remodeling in DCM. Our study implied that the inhibition of Tregs could be the underline mechanism mediating the cardiac insults exacerbated by KB. Furthermore, we revealed that the reduction of MAMs and mitochondrial respiration, which was caused by the blunt IL-33/ST2L signaling, could be the reason of KB-induced Treg suppression.

The current study did not support the regular and wide adoption of KD in diabetes patients. Despite KD being able to confer potential benefits in metabolic indices in this situation, KD, in fact, impaired heart function. Our results were supported by a recent population-based study, which concluded a deleterious result on the coronary artery conferred by low-carbohydrate diet that is similar to KD [3]. However, the findings of the current study supporting the unflavored role of KD in diabetes is sharply in contrast to the previous study, showing beneficial effects of KD in cardiomyocyte survival and interstitial fibrosis [4]. The differences of animal characteristics could be the most plausible explanation of this discrepancy. The hallmark of typical DCM, especially during the early stage, is the impairment of diastolic dysfunction without concurrent systolic defects [19]. However, the abovementioned study showed a significant reduction of LVEF in db/db mice [4], which implied a late stage of DCM or other coexistent causes of cardiac insults. The serum insulin levels also supported the distinct characteristics of the animal used in our and the previous study. Levels of serum insulin were dramatically decreased in db/db mice in the study by Guo et al. [4], while it is well acknowledged that hyperinsulin is one of the basic characteristics of db/db mice as found in the current and many other studies [19, 29, 30], which is the basic pathophysiological alterations of T2DM. Therefore, the animal used in the current study, which reflects the canonical T2DM leading to cardiac remodeling, is distinct from the previous one and this could cause different responses to KD treatment. Besides, it should be noted that treatment was started earlier in the current study (6 weeks vs. 8 weeks) and last for a longer time (12 weeks vs. 8 weeks). Thus, the influence of therapeutic duration could not be neglected since KD could correct metabolic dysfunction, which may confound its intrinsic effects on the heart and longer time may be needed to investigate the real and long-time effects of KD. Other studies reported both beneficial and detrimental effects of KD on the heart [5, 6]. *In vitro* study showed an unflavored role of KD in cardiac fibroblast activation [6] and prevention of cardiomyocyte loss [5]. Besides, KD is also found to be related to live fibrosis [31]. These studies indicated a complex effect of KD, and the adoption of this kind of diet should be cautious.

T cell function may be a key and novel modulator during the development of T2DM and DCM. Interestingly, T cells were also closely related to cardiomyopathy, especially cardiac fibrosis. Alcaide et al. reported that during the development of myocardial infarction, T cells played a central role in the process of myocardial scarring after infarction and directly modulated the phenotypes and functions of cardiac fibroblasts [32]. Furthermore, T cells within the heart could direct the remote fibrosis and scarring in the left ventricle during the chronic remodeling process [32]. Similar to our current findings, one study has reported a reduced circulating CD4^+^CD25^+^ T cell proportion and a suppression of Foxp3 expression in high-fat diet-induced DCM [33]. We discovered that the subset of Tregs, namely, those with ST2L expressing on the membrane, was dramatically reduced upon KD treatment. ST2L^+^ Tregs are highly activated and robust in function, especially in the presence of IL-33, the only discovered ligand of ST2L [34, 35]. On the other hand, the activated Tregs are an important source of IL-33 production. Therefore, it may be plausible to infer that KD treatment blunt IL-33/ST2L signaling result in a positive feedback loop maintaining Treg suppression and contribute to cardiac fibrosis.

MAMs are specific subcellular structure charging calcium, lipid, and metabolite exchange between the endoplasmic reticulum and mitochondria, which are crucial to proper mitochondrial function. It is well known that MAMs are closely connected with cardiovascular diseases. Ikeda et al. revealed that the suppression of mitochondrial dynamics resulted in diminished survival, cardiac hypertrophy, and fibrosis in mice [36]. Moreover, Giorgi et al. found that MAMs promoted the development of infarctions and myocardial fibrosis via recruiting NLRP3 protein and mediating the activation of NLRP3 inflammasome [37]. Besides, MAMs are essential for proper mitochondrial respiration [38]. Some researchers found that excessive Ca^2+^ transfer via MAMs promoted ROS generation and oxidative stress and augmented MAM formation could lead to elevated Ca^2+^ transfer from the ER to mitochondria, resulting in enhanced oxidative stress and mtROS overgeneration [39]. Data on the changes of MAMs in Tregs were scarce but most studies investigating MAMs reported reduction of MAMs, which contributed to disease development in diabetes [40, 41] while reinforcement of MAM conferred protective effects [41]. However, excessive MAMs also impaired mitochondrial oxidative capacity [42]..On the other hand, it should be noted that despite alterations of MAMs being able to explain the reduction of mitochondrial respiration in the current study, MAM reduction did not necessary directly induce mitochondria loss [43]. Therefore, the potential mechanisms leading to mitochondria reduction in response to KB treatment and more studies are merited.

Some limitations of the current study should be acknowledged. Firstly, we did not explore the effects of KD on other key cell types closely related to the development of DCM. As for T cells, Goldberg et al. demonstrated that KD expanded metabolically protective *γδ* T cells and suppressed inflammatory responses [44]. In the cardiomyocytes, You et al. found that KD contributes to the profibrotic actions in fibroblasts possibly via the mTOR signaling pathway in hypertension [6]. Besides, diabetes could modulate a wide range of cellular processes (e.g., autophagy, oxidative stress, and ferroptosis) in cardiomyocytes and endothelial cells, which could be important in the regulations of fibrosis during DCM development [45, 46]. A lack of evidence on these aspects has restricted us to provide full information assessing the way that KD regulates DCM *in vivo*. Secondly, despite the current study focus on DCM and animal model is a canonical DCM, based on the sharp contrast conclusion induced by KD as described above, the conclusions of the current study could be interpreted cautiously if extended to the field outside DCM.

Despite limitations, the current study has explicitly showed an unfavorable effect of KD on ventricular function and cardiac remodeling during the development of DCM, which is partly mediated by regulatory T cell modulation due to altered IL-33/ST2L signaling. There is a great need for studies exploring the effects of KD on other crucial cell types (e.g., cardiac fibroblast and cardiomyocyte) to reveal the molecular mechanism of DCM development. Besides, studies investigating the role of KD on other cardiovascular disorders are also merited to provide a full landscape of KD adoption and cardiovascular outcomes.

## Figures and Tables

**Table 1:** Nutritional composition of ketogenic diet (g/1000 g).

Ingredients	
Vitamins, mixed	10.0
Choline	—
Minerals, mixed	35.0
Fibers	50.0
Sucrose	—
Casein	200.0
Maize starch	—
Soya oil	—
Maize oil	102.0
Lard	424.7
Margarine	178.3
Total	1000

**Table 2:** Metabolic indices of mice.

	m/m	db/db	db/db+KD	db/db+PBS	*P* value
Body weight (g)	28.72 ± 3.72	40.12 ± 3.47^∗^	43.48 ± 3.66^#^	34.26 ± 5.87	<0.01
Heart weight (g)	0.26 ± 0.02	0.41 ± 0.03^∗^	0.46 ± 0.03^¶^	0.393 ± 0.042	<0.01
Fasting glucose (mM)	10.06 ± 0.57	23.01 ± 3.56^∗^	17.43 ± 3.15^¶^	23.11 ± 3.79	<0.01
TC (mg/dl)	216.37 ± 15.37	240.58 ± 20.75	225.48 ± 35.05	234.57 ± 20.59	0.35
TG (mg/dl)	116.13 ± 10.35	132.68 ± 12.84	114.25 ± 15.48^§^	135.54 ± 12.84	0.02
LDL-c (mg/ml)	153.59 ± 5.78	157.15 ± 8.39	152.89 ± 10.60	154.99 ± 13.67	0.89
Fasting insulin (mIU/l)	21.59 ± 4.60	49.333 ± 9.522^∗^	31.47 ± 6.36^#^	50.32 ± 11.94	<0.01
HOMA-IR	9.70 ± 2.30	49.83 ± 8.60^∗^	24.35 ± 6.68^#^	52.30 ± 17.57	<0.01

KD: ketogenic diet; PBS: phosphate-buffered solution; TC: total cholesterol; TG: triglyceride; LDL-c: low-density lipoprotein cholesterol; HOMA-IR: homeostasis model assessment-insulin resistance; ^∗^compared with m/m, *P* < 0.01; ^#^compared with db/db+PBS, *P* < 0.01; ^¶^compared with db/db+PBS, *P* < 0.05; ^§^compared with db/db+PBS, *P* < 0.05; *P* values were derived from ANOVA.

## Data Availability

The authors declare that all data supporting the findings of this study are available within the paper.
